# Daidzein Inhibits Human Platelet Activation by Downregulating Thromboxane A_2_ Production and Granule Release, Regardless of COX-1 Activity

**DOI:** 10.3390/ijms241511985

**Published:** 2023-07-26

**Authors:** Hyun-Jin Hong, Gi-Suk Nam, Kyung-Soo Nam

**Affiliations:** 1 Department of Pharmacology and Intractable Disease Research Center, School of Medicine, Dongguk University, Gyeongju 38066, Republic of Korea; hyunjin5886@dongguk.ac.kr; 2 Department of Biomedical Laboratory Science, Honam University, 120, Honamdae-gil, Gwangsan-gu, Gwangju 62399, Republic of Korea

**Keywords:** daidzein, cardiovascular disease, isoflavone, platelet activation, thromboxane A_2_

## Abstract

Platelets play crucial roles in cardiovascular diseases (CVDs) by regulating hemostasis and blood coagulation at sites of blood vessel damage. Accumulating evidence indicates daidzein inhibits platelet activation, but the mechanism involved has not been elucidated. Thus, in this study, we investigated the mechanism responsible for the inhibition of collagen-induced platelet aggregation by daidzein. We found that in collagen-induced platelets, daidzein suppressed the production of thromboxane A_2_ (TXA_2_), a molecule involved in platelet activation and aggregation, by inhibiting the cytosolic phospholipase A_2_ (cPLA_2_) signaling pathway. However, daidzein did not affect cyclooxygenase-1 (COX-1). Furthermore, daidzein attenuated the PI3K/PDK1/Akt/GSK3αβ and MAPK (p38, ERK) signaling pathways, increased the phosphorylation of inositol trisphosphate receptor1 (IP_3_R1) and vasodilator-stimulated phosphoprotein (VASP), and increased the level of cyclic adenosine monophosphate (cAMP). These results suggest that daidzein inhibits granule release (ATP, serotonin, P-selectin), integrin α_IIb_β_3_ activation, and clot retraction. Taken together, our study demonstrates that daidzein inhibits collagen-induced platelet aggregation and suggests that daidzein has therapeutic potential for the treatment of platelet aggregation-related diseases such as atherosclerosis and thrombosis.

## 1. Introductions

Cardiovascular disease (CVD) is a major, growing problem worldwide. Platelet-related cardiovascular disease refers to conditions in which abnormalities in platelet function or excessive platelet activation contribute to the development and progression of cardiovascular disorders. Platelets are small, disc-shaped cells in the blood that play a crucial role in blood clotting. Normal platelets play a critical role in preventing blood loss when blood vessels are damaged. While platelets are necessary for wound healing and preventing excessive bleeding, excessive or abnormal platelet aggregation is a contributory factor in CVDs such as thrombosis, stroke, and atherosclerosis [1,2,3].

Platelets are metabolically active cells that lack a nucleus and exist as small cellular fragments containing numerous functional organelles, including endoplasmic reticulum, Golgi apparatus, and mitochondria [4]. Signaling within platelets occurs after receptors on platelet surfaces are activated by agonists such as collagen, thrombin, and adenosine diphosphate (ADP) [5]. When blood vessels are injured, platelet agonists such as collagen, ADP, and thrombin are exposed or produced locally at sites of injury. In addition, the platelet glycoprotein VI (GPVI) receptor-mediated signaling pathway primarily responds to collagen-induced platelet activation [6,7].

Granule release is a process that occurs in platelets during platelet activation. Platelets contain different types of granules, including dense granules and alpha granules, which store various bioactive molecules [8]. Dense granules release molecules such as serotonin, ADP, calcium, and adenosine triphosphate (ATP). These molecules play a role in promoting platelet aggregation and blood clot formation. They act by activating nearby platelets and enhancing the recruitment of additional platelets to the site of injury [9]. Alpha granules in platelets contain various proteins, one of which is P-selectin. P-selectin is translocated to the platelet surface upon activation. Its interaction with P-selectin glycoprotein ligand-1 facilitates leukocyte adhesion and recruitment, contributing to immune response and inflammation. Additionally, P-selectin is involved in platelet–platelet interactions and platelet aggregation, playing a role in hemostasis and thrombus formation [10,11].

When platelets are activated, cytosolic phospholipase A_2_ (cPLA_2_) releases arachidonic acid (AA) from cell membrane phospholipids. AA can then be converted into various inflammatory mediators by enzymes such as cyclooxygenase-1 (COX-1) [12]. COX-1 catalyzes the conversion of arachidonic acid into prostaglandin H_2_, which is subsequently converted to thromboxane A_2_ (TXA_2_) by thromboxane synthase (TXAS). TXA_2_ promotes platelet aggregation and vasoconstriction, contributing to the formation of blood clots [13].

The cyclic adenosine monophosphate (cAMP) signaling pathway influences multiple aspects of platelet function, including the regulation of vasodilator-stimulated phosphoprotein (VASP), inositol trisphosphate receptor (IP_3_R), and integrin activation [14]. IP_3_R is an intracellular calcium channel that is involved in calcium signaling. When platelets are activated by agonists, IP_3_R is stimulated, resulting in the release of calcium ions from intracellular stores. This calcium release is critical for platelet activation and aggregation [15]. On the other hand, phosphorylated VASP has been shown to promote the separation of the α and β subunits of integrin α_IIb_β_3_, leading to integrin activation and enhanced binding to ligands such as fibrinogen. Integrins are a family of cell surface receptors that mediate cell–cell and cell–extracellular matrix interactions. In platelets, integrin α_IIb_β_3_ is primarily responsible for platelet aggregation by binding to fibrinogen and other ligands [16,17]. Activation of integrin α_IIb_β_3_ is crucial for platelet aggregation and clot formation [18].

When platelets are stimulated by agonists like thrombin or collagen, the phosphoinositide 3-kinase (PI3K)/Akt pathway is activated. PI3K phosphorylates phosphatidylinositol 4,5-bisphosphate to generate phosphatidylinositol 3,4,5-trisphosphate (PIP_3_). PIP_3_ acts as a second messenger and recruits Akt to the plasma membrane, where it is activated by phosphorylation [19]. Activated Akt then initiates a cascade of downstream events that contribute to platelet activation. It phosphorylates and activates various targets including proteins involved in granule release and integrin α_IIb_β_3_ activation. These processes promote platelet aggregation, adhesion, and clot formation [20,21].

**Figure 1:**
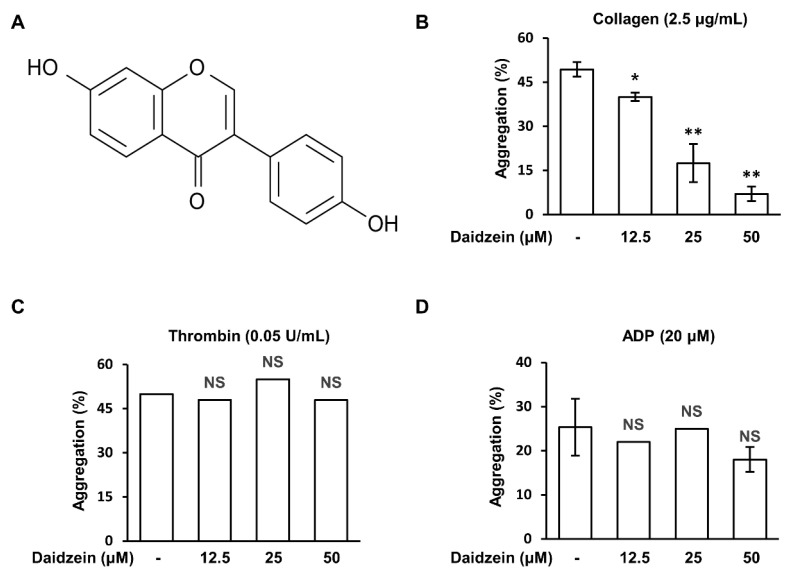
Effects of daidzein on various agonist-induced platelet aggregations. (**A**) Chemical structure of daidzein. (**B**) Effect of daidzein on collagen (2.5 μg/mL)-induced platelet aggregation. (**C**) Effect of daidzein on thrombin (0.05 U/mL)-induced platelet aggregation. (**D**) Effect of daidzein on ADP (20 μM)-induced platelet aggregation. Results are presented as means ± SDs (n = 3). * *p* < 0.05, ** *p* < 0.001 versus collagen-treated platelets. NS, not significant versus agonist-treated platelets.

Isoflavones, which are abundant in soybeans, have garnered considerable attention from the scientific community [22]. Early studies have shown that isoflavones might exert anti-platelet function through cAMP regulation, tyrosine kinase, calcium messenger, and TxA_2_ pathway inhibition [23,24,25,26,27]. Genistein, a tyrosine kinase inhibitor, is known to inhibit thromboxane-mediated platelet activation, and daidzein, which has a similar chemical formula, is also known to have anti-platelet effects [25]. Daidzein is a type of isoflavone, a group of compounds found in plants, including soybeans (Figure 1A). Daidzein has been reported to play a significant role in the prevention and treatment of a variety of diseases such as cancer, CVD, diabetes, osteoporosis, skin disease, and neurodegenerative disease [28]. However, the mechanism responsible for its anti-platelet effects has not been fully elucidated, and thus we investigated this mechanism.

## 2. Results

### 2.1. Effects of Daidzein on Human Platelet Aggregation induced by Different Agonists

To confirm the effect of daidzein on human platelet aggregation, we conducted a human platelet aggregation test using PRP and washed platelets. Daidzein (12.5–50 μM) inhibited collagen (2.5 μg/mL)-induced platelet aggregation in a concentration-dependent manner (Figure 1B) but had no inhibitory effect on thrombin (0.05 U/mL) or ADP (20 μM)-induced platelet aggregation (Figure 1C,D).

### 2.2. Effect of Daidzein on Granule Release by Collagen-Activated Platelets

**Figure 2:**
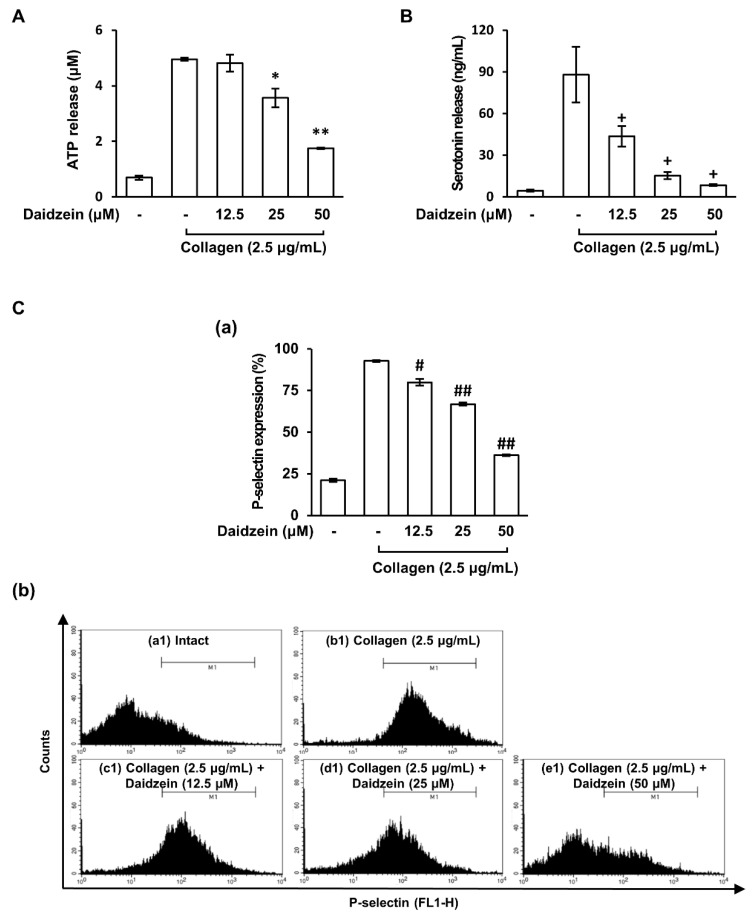
Effects of daidzein on granule release by collagen-induced platelets. (**A**) Effect of daidzein on ATP release. (**B**) Effect of daidzein on serotonin release. (**C**) (**a**) Flow cytometry histograms of collagen-induced P-selectin expressions. (**b**) Effect of daidzein on collagen-induced P-selectin expressions. The methods used to assess ATP, serotonin, and P-selectin expressions are described in Materials and Methods. Results are presented as means ± SDs (n = 3). * *p* < 0.05, ** *p* < 0.001 versus collagen-induced platelets. + *p* < 0.05 versus collagen-induced platelets. # *p* < 0.05, ## *p* < 0.001 versus collagen-induced platelets.

Activated platelets release alpha granules and dense granules which play important roles in hemostasis [9]. In our study, daidzein exhibited an inhibitory effect on collagen-induced platelet aggregation. Therefore, we investigated the effect of daidzein on granule release in collagen-induced platelets. Figure 2A,B show that when platelets were stimulated with collagen (2.5 µg/mL), ATP and serotonin release increased, but pretreatment with daidzein (12.5–50 µM) significantly and dose-dependently inhibited these releases. Furthermore, daidzein significantly reduced the collagen-induced surface expression of P-selectin (Figure 2C). These results suggest that daidzein inhibits collagen-induced platelet aggregation by suppressing granule release.

### 2.3. Daidzein Inhibited Thromboxane A_2_ Production Regardless of COX-1 Activity in Collagen-Induced Platelets

**Figure 3:**
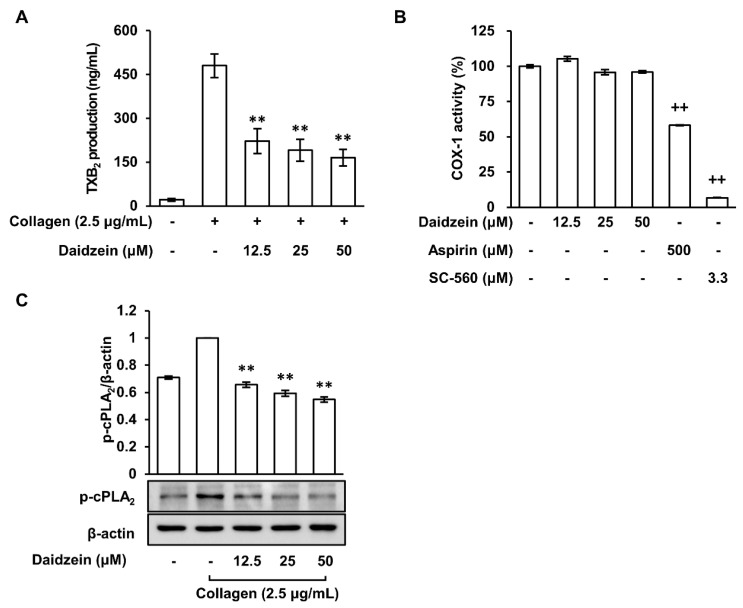
Effects of daidzein on TXB_2_ production, COX-1 activity, and cPLA_2_ phosphorylation in collagen-activated platelets. (**A**) Effect of daidzein on TXB_2_ production. (**B**) Effect of daidzein on COX-1 activity. (**C**) Effect of daidzein on cPLA_2_ phosphorylation. Measurement of TXB_2_ production, COX-1 activity, Western blotting analysis were described in Materials and Methods. Results are presented as means ± SDs (n = 3). ** *p* < 0.001 versus collagen-induced platelets, ++ *p* < 0.001 versus COX-1 positive control.

Platelet aggregation plays a key role in the development of atherosclerosis and thrombosis, and TXA_2_ stimulates this process [13]. In our study, the production of TXA_2_ was indirectly verified using its stable metabolite TXB_2_. The production of TXB_2_ increased when platelets were treated with collagen (2.5 μg/mL), but pretreatment with daidzein (12.5–50 μM) significantly attenuated the production of TXB_2_ (Figure 3A). In platelets, COX-1 is essential for the synthesis of TXA_2_ [29]. However, our results show that daidzein (12.5–50 μM) had no inhibitory effect on COX-1 activity (Figure 3B), whereas the positive controls, aspirin (500 μM) and SC-560 (3.3 μM) (a COX-1 inhibitor), both reduced COX-1 activity. These results suggest that daidzein inhibits TXA_2_ production through a mechanism other than COX-1 activity.

### 2.4. Inhibitory Effect of Daidzein on the Phosphorylation of Cytosolic Phospholipase A_2_

cPLA_2_ releases arachidonic acid, COX-1 converts arachidonic acid into prostaglandin H_2_, and TXA_2_ is derived from prostaglandin H_2_, playing a role in platelet aggregation and vasoconstriction [30]. Therefore, we investigated the effect of daidzein on the phosphorylation of cPLA_2_, an upstream signaling pathway that regulates the production of TXB_2_. When platelets were treated with collagen (2.5 μg/mL), cPLA_2_ phosphorylation increased (Figure 3C). However, this increase was attenuated in the presence of daidzein (12.5–50 μM). These results suggest that daidzein inhibits the phosphorylation of cPLA_2_ rather than COX-1 activity, thereby inhibiting TXB_2_ production in collagen-induced platelet aggregation.

### 2.5. Effects of Daidzein on Cyclic Adenosine Monophosphate Levels and Phosphodiesterase Activity in Collagen-Induced Platelets

**Figure 4:**
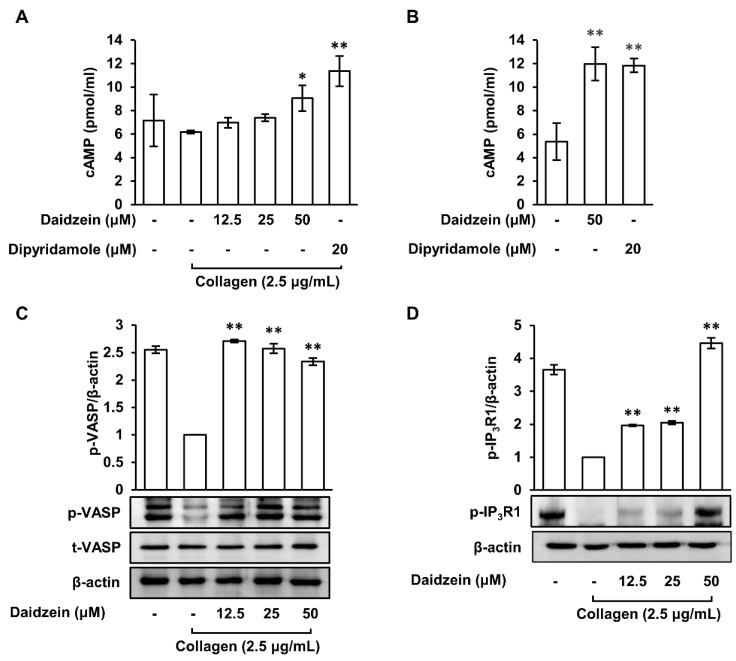
Effects of daidzein on cAMP levels and cAMP-dependent phosphoproteins. (**A**) Effect of daidzein on cAMP level in collagen-treated platelets. (**B**) Effect of daidzein on phosphodiesterase in unstimulated platelets. (**C**) Effect of daidzein on VASP (Ser^157^) phosphorylation. (**D**) Effect of daidzein on IP_3_R1 phosphorylation. Measurement of cAMP level and Western blotting analysis were described in Materials and Methods. Results are presented as means ± SDs (n = 3). * *p* < 0.05, ** *p* < 0.001 versus collagen-induced platelets.

cAMP is an important mediator of platelet activity and inhibits platelet aggregation [31]. Thus, we assessed the effects of daidzein on cAMP levels after collagen-induced platelet aggregation. When pretreated with daidzein (12.5–50 μM), cAMP levels significantly and dose-dependently increased versus collagen (Figure 4A). Furthermore, pretreatment with dipyridamole (20 μM), a phosphodiesterase (PDE) inhibitor and positive control, also increased cAMP levels versus collagen. Platelets contain several PDEs that catalyze the degradation of cAMP, a second messenger that regulates platelet activation [5]. The activity of PDE was indirectly confirmed whether daidzein inhibits the activity of PDE, an enzyme that degrades cAMP, by measuring the level of cAMP in not stimulated with collagen. Figure 4B shows that daidzein (50 μM) and dipyridamole (20 μM) both increased cAMP levels in unstimulated platelets. These results suggest that daidzein increases cAMP levels by inhibiting cAMP hydrolysis by attenuating PDE activity.

### 2.6. Effects of Daidzein on the Phosphorylations of VASP (Ser^157^) and IP_3_R1 in Collagen-Activated Platelets

Increasing cAMP levels causes protein kinase A (PKA) activation, and activated PKA causes the phosphorylations of VASP and IP_3_R [14]. Therefore, we investigated the effects of daidzein on the phosphorylations of VASP (Ser^157^) and IP_3_R1. The results showed that the phosphorylations of VASP (Ser^157^) and IP_3_R1 were reduced by collagen (2.5 μg/mL). However, when platelets were pretreated with daidzein (12.5–50 μM) and then with collagen, the phosphorylations of VASP (Ser^157^) and IP_3_R1 were increased as compared with collagen-treated platelets (Figure 4C,D). These results suggest that daidzein increases the phosphorylations of VASP (Ser^157^) and IP_3_R1 by increasing cAMP levels.

### 2.7. Effect of Daidzein on Fibrinogen Binding to Integrin α_IIb_β_3_ on Collagen-Induced Platelets

**Figure 5:**
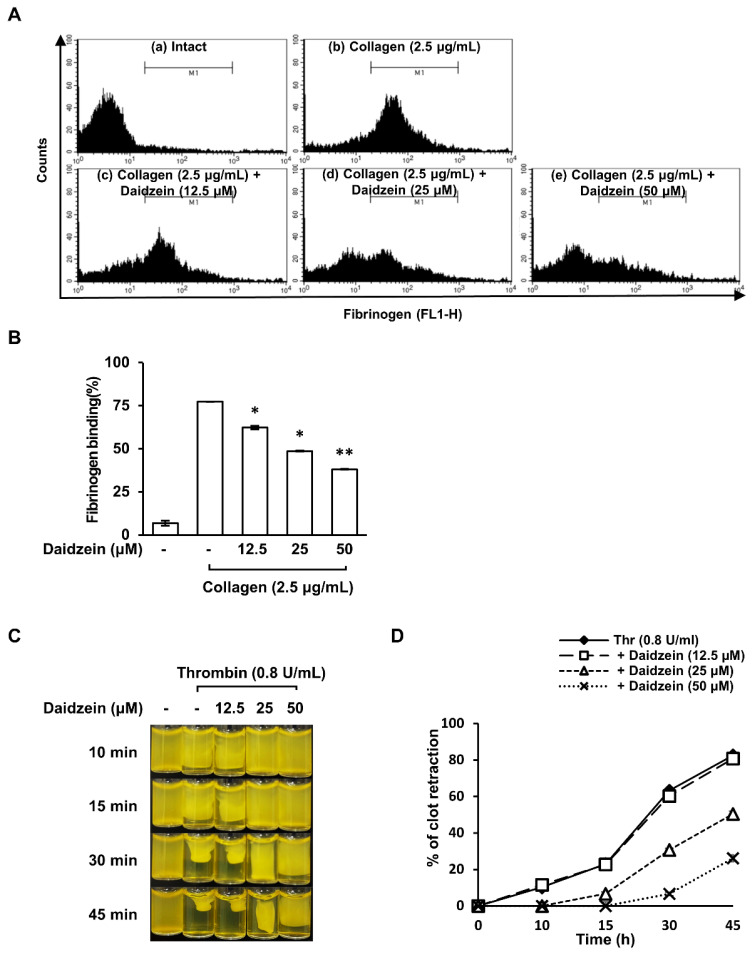
Effects of daidzein on fibrinogen binding to integrin α_IIb_β_3_ and clot retraction. (**A**) Flow cytometry histograms of collagen-induced fibrinogen binding to integrin α_IIb_β_3_. (**B**) Effect of daidzein on collagen-induced fibrinogen binding to integrin α_IIb_β_3_. (**C**) Photographs showing the inhibitory effect of daidzein on thrombin-induced fibrin clot retraction. (**D**) Effect of daidzein on thrombin-induced fibrin clot retraction. Fibrinogen binding to integrin α_IIb_β_3_ and clot retraction were assessed as described in Materials and Methods. Results are presented as means ± SDs (n = 3). * *p* < 0.05, ** *p* < 0.001 versus collagen-induced platelets.

Activated integrin α_IIb_β_3_ can bind to fibrinogen and act as a molecular bridge between adjacent platelets, thereby facilitating platelet–platelet interactions [32]. Therefore, we investigated the effect of daidzein on fibrinogen binding to integrin α_IIb_β_3_, because daidzein increased the phosphorylation (inactive form) of VASP (Ser^157^). Figure 5A shows the fluorescence signal of Alexa Fluor 488-conjugated fibrinogen to integrin α_IIb_β_3_ binding in the presence of daidzein (12.5–50 μM). Collagen (2.5 μg/mL)-activated platelets exhibited integrin α_IIb_β_3_ activation versus unstimulated platelets. However, daidzein (12.5–50 μM) pretreatment significantly attenuated collagen-induced integrin α_IIb_β_3_ activation (Figure 5A,B). These results suggest that daidzein suppresses platelet aggregation by increasing cAMP levels and attenuating integrin α_IIb_β_3_ activation.

### 2.8. Inhibitory Effect of Daidzein on Clot Retraction

Outside-in signaling through integrin α_IIb_β_3_ plays a critical role in clot retraction due to fibrinogen binding [33]. Therefore, we investigated the effect of daidzein on clot retraction after stimulating platelets with thrombin (0.05 U/mL). Clot retraction was observed when PRP was treated with thrombin (0.05 U/mL), and this was significantly inhibited by daidzein pretreatment (12.5–50 μM) (Figure 5C,D). These results suggest that daidzein inhibits platelet-induced clot retraction by inhibiting the activation of integrin α_IIb_β_3_.

### 2.9. Inhibitory Effects of Daidzein on the Phosphorylations of PI3K/PDK1/Akt/GSK3αβ and MAPK (p38 and ERK) Pathways

**Figure 6:**
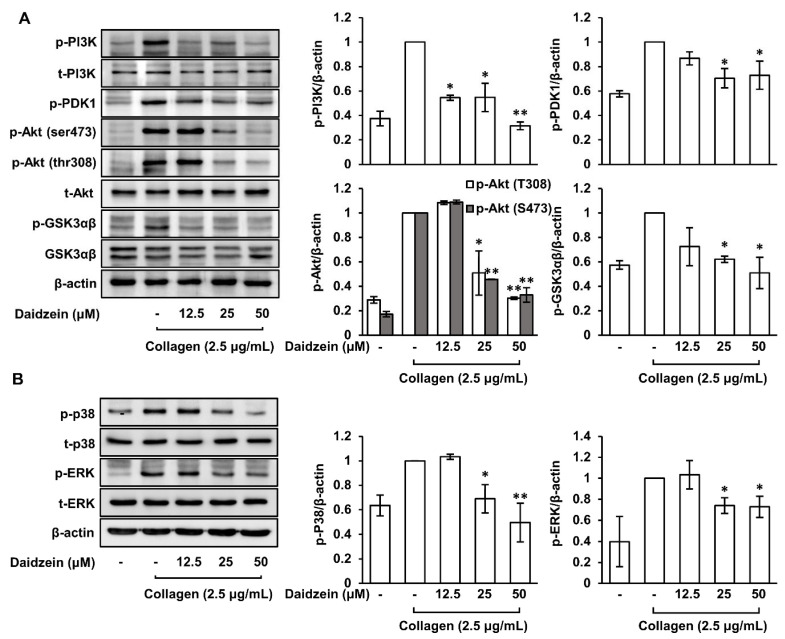
Effects of daidzein on PI3K/PDK1/Akt/GSK3αβ and MAPK (p38 and ERK) pathways. (**A**) Effects of daidzein on the phosphorylations of the PI3K, PDK1, Akt, GSK3αβ, and density in collagen-induced platelets. (**B**) Effects of daidzein on the phosphorylation of MAPKs (p38 and ERK) and density in collagen-induced platelets. Western blotting analysis was performed as described in Materials and Methods section. Results are presented as means ± SDs (n = 3). * *p* < 0.05, ** *p* < 0.001 versus collagen-induced platelets.

The PI3K/Akt pathway plays an important role in collagen-induced platelet aggregation as it phosphorylates and activates various targets including proteins involved in granule release and the activation of integrin α_IIb_β_3_ [20,21]. Therefore, we investigated daidzein-mediated phosphorylation changes in the PI3K/PDK1/Akt/GSK3αβ pathways in collagen-induced platelets and found that daidzein pretreatment (12.5–50 μM) significantly suppressed their phosphorylations (Figure 6A). The MAPK pathway promotes platelet activation and aggregation by regulating processes involved in platelet function such as the activation of integrins and the production of TXA_2_ [34]. Our results show that daidzein significantly attenuated the phosphorylations of MAPK (p38, ERK) pathways in collagen-induced platelets (Figure 6B).

### 2.10. The Synergistic Effect of Daidzein and Aspirin

**Figure 7:**
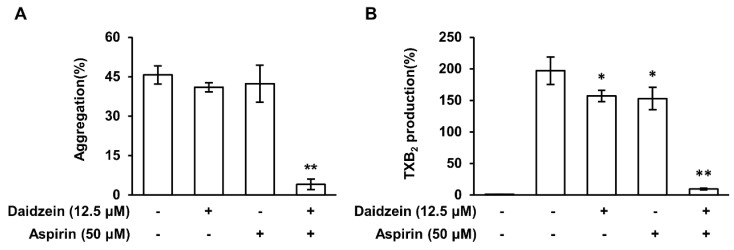
The synergistic effect of daidzein plus aspirin. (**A**) Effects of daidzein and aspirin cotreatment on collagen-induced platelet aggregation. (**B**) Effects of cotreatment on collagen-mediated TXB_2_ production. Results are presented as means ± SDs (n = 3). * *p* < 0.05, ** *p* < 0.001 versus collagen-induced platelets.

We found that daidzein inhibited collagen-induced platelet aggregation and thrombin-induced clot retraction without affecting the COX-1 pathway. Therefore, we confirmed the synergistic effect when aspirin, a COX inhibitor, was treated together with daidzein in a low concentration. Figure 7A shows that cotreatment with daidzein (12.5 μM) and aspirin (50 μM) strongly inhibited platelet aggregation at these low concentrations, whereas, when treated individually, daidzein and aspirin weakly inhibited platelet aggregation. Furthermore, daidzein and aspirin cotreatment at these low concentrations inhibited the production of TXB_2_ in collagen-stimulated platelets (Figure 7B). These results suggest that the side effects of COX-1 inhibition by aspirin can be reduced by daidzein coadministration.

## 3. Discussion

CVD is a group of disorders that includes coronary artery disease, heart failure, arrhythmias, and stroke [35], all of which affect the heart and blood vessels. Platelet aggregation is an important process during the development of CVD. Platelets are small blood cells that play a crucial role in blood clotting, which is essential to stop bleeding after injury. However, under certain circumstances, platelets can become activated and clump together to form blood clots that can block blood vessels [36]. This process is known as platelet aggregation and is a critical component of atherosclerotic plaque formation, which is the primary cause of coronary artery disease. In addition to contributing to the formation of atherosclerotic plaque, platelet aggregation can also result in blood clots in the arteries that supply blood to the heart (coronary thrombosis) and cause heart attacks. Platelet aggregation can also contribute to the development of other types of CVD, including stroke [37,38].

**Figure 8:**
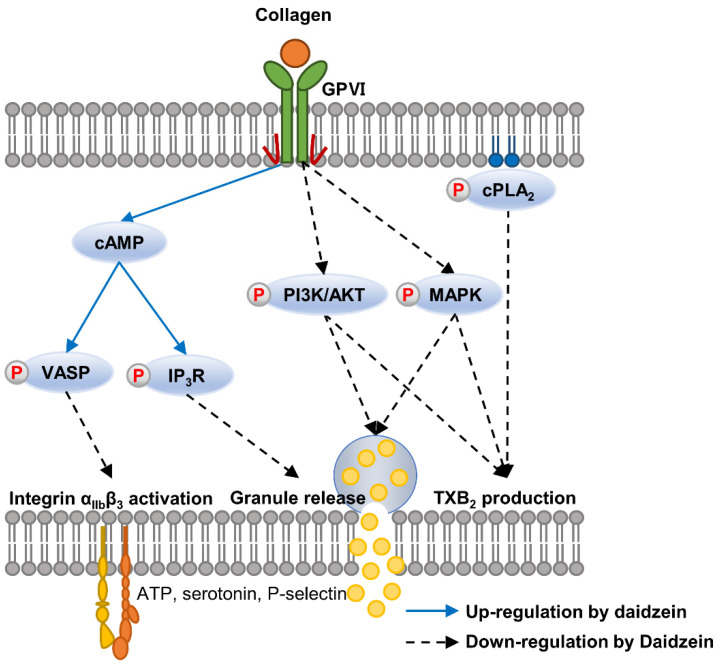
A schematic of the inhibitory effects of daidzein on collagen-mediated intracellular signaling pathways.

Daidzein is an isoflavone found in soy products and various plants and has been reported to have several health benefits, including anti-inflammatory and antioxidant effects [28]. Furthermore, evidence suggests that daidzein might inhibit platelet activation, although the precise mechanism is not fully understood. In this study, we sought to reveal the mechanism responsible for the anti-platelet effect of daidzein. We found that daidzein had inhibitory effects on collagen-induced platelet aggregation, granule release, TXA_2_ production, integrin α_IIb_β_3_ activation, and clot retraction, and that it increased cAMP levels and the phosphorylations of VASP (Ser^157^) and IP_3_R1 but attenuated the phosphorylations of PI3K/PDK1/Akt/GSK3αβ and MAPKs (p38 and ERK) (Figure 8).

Activation leads to the release of alpha granules, dense granules, and lysosomes by platelets [39]. Alpha granules contain a variety of proteins, such as fibrinogen, von Willebrand factor, and platelet-derived growth factor, which are important for platelet function and wound healing [10]. Dense granules contain small molecules, such as ADP and serotonin, which are released upon platelet activation and contribute to platelet aggregation and blood clot formation [8]. We found that daidzein attenuated the release of granules (ATP, serotonin, and P-selectin), which suggests daidzein inhibits platelet activation.

When platelets are activated, they activate cPLA_2_, which acts on phospholipids in the cell membrane to release AA. This released AA is then converted into prostaglandins and thromboxane by COX enzymes. Furthermore, thromboxane is synthesized in platelets by TXAS and is involved in platelet aggregation and clot formation [13]. Our results show that daidzein did not affect COX-1 but inhibited thromboxane production by suppressing cPLA_2_ phosphorylation.

cAMP acts as a second messenger and inhibits platelet activation, whereas PDEs are responsible for the breakdown of cAMP to AMP [40]. The present study indirectly demonstrates that daidzein increases cAMP levels by inhibiting PDE activity, which suggests a potential mechanism for its inhibitory effect on platelet activation. Furthermore, in collagen-induced platelets, daidzein increased the phosphorylations of two cAMP-dependent proteins, VASP and IP_3_R1, which are regulated by cAMP and play key roles in the regulation of platelet function [31].

VASP plays a key role in clot retraction, that is, the process by which blood clots are compacted and strengthened. During clot retraction, platelets change shape and pull on fibrin strands to reduce clot sizes [41]. Integrins are transmembrane proteins involved in platelet adhesion and aggregation and mediate interactions between platelets and the extracellular matrix and, thus, play important roles in platelet function and clot formation [16,18]. The regulations of VASP and integrin α_IIb_β_3_ are critical for maintaining hemostasis and preventing thrombotic disorders because they play important roles in platelet function, including clot retraction. Our study shows that daidzein-induced increases in cAMP level and VASP (Ser^157^) phosphorylation are closely related to the inhibition of integrin α_IIb_β_3_ activation. Furthermore, our findings indicate that the inhibitory effect of daidzein on integrin activity has an impact on clot retraction.

The PI3K/Akt and MAPK pathways are two important signaling pathways involved in platelet activation and aggregation. The PI3K/Akt pathway is activated in response to various platelet agonists, including thrombin, collagen, and ADP, and promotes platelet activation, granule release, and integrin activation [32,42]. The MAPK pathway is also activated in response to various platelet agonists and is composed of three main components, namely, ERK, c-Jun N-terminal kinase (JNK), and p38 MAPK. When activated, this pathway promotes platelet activation and aggregation by regulating the expression of genes involved in platelet function, such as integrins and TXA_2_ [43,44]. We observed that daidzein suppressed the phosphorylations of PI3K, PDK1, Akt (Ser^473^ and Thr^308^), GSK3αβ, p38, and ERK in collagen-induced platelets, which suggests that the anti-platelet effect of daidzein might be due to the inhibition of integrin α_IIb_β_3_ activation, TXA_2_ production, and granule release, which are regulated by these pathways.

Aspirin is an anti-platelet drug and COX inhibitor. However, because it inhibits COX-1 and COX-2, aspirin can cause side effects due to the suppression of COX-1. Currently, numerous studies are being conducted to minimize the adverse effects associated with COX-1 inhibition [45]. Interestingly, we found that daidzein had no effect on COX-1 activity and observed a synergistic effect on platelet aggregation and TXA_2_ production when daidzein and aspirin were cotreated at low concentrations. Notably, our results suggest that daidzein may be used to reduce aspirin dosages, potentially decreasing the severity and incidence of side effects caused by COX-1 inhibition.

## 4. Materials and Methods

### 4.1. Materials

Collagen, thrombin, and ADP were obtained from Chrono-Log Co. (Havertown, PA, USA). Daidzein and aspirin were purchased from Sigma Aldrich (St. Louis, MO, USA). The thromboxane B_2_ (TXB_2_) ELISA, cyclic AMP ELISA, and COX fluorescent inhibitor screening assay kits, and dipyridamole, were acquired from Cayman Chemical (Ann Arbor, MI, USA). CytoTox 96^®^ Non-Radioactive Cytotoxicity Assay was purchased from Promega (Madison, WI, USA), and the serotonin assay kit was purchased from Abnova (Taipei, Taiwan). Fura-2/acetoxymethyl ester (AM) and Alexa Fluor 488-conjugated fibrinogen were obtained from Molecular Probes (Eugene, OR, USA). Alexa Fluor 488 anti-human CD62P (P-Selectin) antibody was purchased from BioLegend (San Diego, CA, USA), and the ATP assay kit was purchased from Biomedical Research Service Center (Buffalo, NY, USA). Protease inhibitor cocktail and phosphatase inhibitor cocktail were procured from GenDEPOT (Barker, TX, USA), and antibodies for p-cPLA_2_, PI3K, phospho-PI3K, phosphoinositide-dependent kinase 1 (PDK1), phosphor-PDK1, protein kinase B (Akt), phospho-Akt (Ser^473^), phospho-Akt (Thr^308^), glycogen synthase kinase-3αβ (GSK3αβ), phospho-GSK3αβ, p38, phospho-p38, extracellular signal-regulated kinase (ERK), phosphor-ERK, VASP, phospho-VASP (Ser^157^), p-IP_3_R1, and β-actin were acquired from Cell Signaling Technology (Beverly, MA, USA). Horseradish peroxidase-conjugated secondary anti-rabbit antibody, anti-mouse antibody, and Pierce™ BCA protein assay kits were purchased from Thermo Fisher Scientific (Waltham, MA, USA), and polyvinylidene fluoride (PVDF) membranes were purchased from Pall Life Sciences (Port Washington, NY, USA).

### 4.2. Preparation of Washed Human Platelets

Human platelet-rich plasma (PRP) was obtained from the Korean Red Cross Blood Center (Daegu, South Korea), centrifuged at 125× *g* for 10 min to remove unwanted cells, and then centrifuged at 1300× *g* for 10 min to obtain platelet pellets. Platelet pellets were then washed twice with washing buffer (138 mM NaCl, 2.7 mM KCl, 12 mM NaHCO_3_, 0.36 mM NaH_2_PO_4_, 5.5 mM glucose, and 1 mM Na_2_ EDTA, pH 6.5) and resuspended in suspension buffer (138 mM NaCl, 2.7 mM KCl, 12 mM NaHCO_3_, 0.36 mM NaH_2_PO_4_, 0.49 mM MgCl_2_, 5.5 mM glucose, 0.25% gelatin, pH 6.9). The above procedures were performed at room temperature (RT) to avoid platelet activation by low temperatures.

### 4.3. Determination of Human Platelet Aggregation

Platelet aggregation was assessed by light-transmission aggregometry (Chrono-Log, Corp., Havertown, PA, USA) at 37 °C. Briefly, washed human platelets (10^8^ cells/mL) were preincubated with various concentrations of daidzein or 0.2% dimethyl sulfoxide (DMSO) for 2 min in the presence of 2 mM CaCl_2_, stimulated with collagen (2.5 μg/mL) or thrombin (0.05 U/mL), and monitored for 5 min. PRP was preincubated with various concentrations of daidzein (12.5–50 μM) or 0.2% DMSO for 2 min, stimulated with ADP (20 μM), and monitored for 5 min. Platelet aggregations were calculated as percentages (%) after the reaction had proceeded for 5 min.

### 4.4. Determination of ATP and Serotonin Release

After platelet aggregation was stopped by adding ice-cold 5 mM EDTA, the supernatant was centrifuged at 2000× *g* for 5 min at 4 °C. Released ATP and serotonin levels were assessed using an ATP assay kit (Biomedical Research Service Center, Buffalo, NY, USA) and a serotonin ELISA kit (Abnova, Taipei, Taiwan) using a SpectraMax M2e microplate reader (Molecular Devices, Sunnyvale, CA, USA).

### 4.5. Determination of TXA_2_ Production

After platelet aggregation was stopped by adding ice-cold 5 mM EDTA, the supernatant was obtained by centrifugation at 2000× *g*. It was then diluted 1:500 and used to assess TXA_2_ production. Since TXB_2_ is a stable metabolite of TXA_2_, TXA_2_ production was assessed using a TXB_2_ EISA kit (Cayman Chemical, Ann Arbor, MI, USA).

### 4.6. Determination of COX-1 Activity

COX-1 activity was evaluated using a COX fluorescent inhibitor screening assay kit (Cayman Chemical, Ann Arbor, MI, USA). The assay was performed in a final volume of 200 μL in black 96-well non-binding microplates. Various concentrations of daidzein (12.5–50 μM) were incubated for 5 min at RT in the following solution: 150 μL of assay buffer (Tris-HCl, 100 mM, pH 8), 10 μL of hemin (final concentration 1 μM), and 10 μL of COX-1; then, 10 μL of 10-acetyl-3,7-dihydroxyphenoxazine (ADHP) was added. The reaction was started by adding AA (10 μL, final concentration of 100 μM). After incubation for 2 min at RT, COX-1 activity was determined using a SpectraMax M2e (Molecular Devices, Sunnyvale, CA, USA). The COX-1 inhibitor SC-560 (3.3 μM) and aspirin (500 μM) were used as positive controls. Three independent experiments were performed in triplicate and results were calculating using:

% inhibition = [initial activity − sample activity]/initial activity × 100(1)

### 4.7. Determination of cAMP Levels

Platelet suspensions (10^8^ cells/mL) were preincubated with various concentrations of daidzein or 0.2% dimethyl sulfoxide (DMSO) for 2 min in the presence of 2 mM CaCl_2_ and then stimulated with collagen (2.5 μg/mL) for 5 min. Ethanol (750 μL, 80%) was then added to prevent platelet aggregation, and samples were reacted for 30 min at RT then separated by centrifugation at 2000× *g* for 10 min at 4 °C. Supernatants were then transferred to new tubes and dried by vacuum centrifugation. The dried pellets obtained were dissolved in ELISA buffer from a cyclic AMP ELISA kit (Cayman Chemical, Ann Arbor, MI, USA), and cAMP levels in samples were determined using the SpectraMax M2e (Molecular Devices, Sunnyvale, CA, USA).

### 4.8. Flow Cytometry Analysis

Washed human platelets were preincubated with various concentrations of daidzein (12.5–50 μM) or 0.2% DMSO for 2 min in the presence of 2 mM CaCl_2_ and stimulated with collagen (2.5 μg/mL) for 5 min. Samples were then fixed with 0.5% paraformaldehyde for 30 min at 4 °C, washed three times with phosphate-buffered saline (PBS), suspended in ice-cold 3% bovine serum albumin (BSA)/PBS, and incubated with Alexa Fluor 488 anti-human CD62P (P-selectin) antibody or Alexa Fluor 488-conjugated human fibrinogen in 3% BSA/PBS for 30 min at 4 °C in the dark. After centrifugation and washing, platelet pellets were dissolved in 3% BSA/PBS. Flow cytometry was performed using a FACS Calibur II (BD Biosciences, San Jose, CA, USA) and CellQuest version 5.2.1.

### 4.9. Clot Retraction Assay

Human PRP was preincubated with various concentrations of daidzein (12.5–50 μM) or 0.2% DMSO for 5 min at 37 °C and induced to coagulate with thrombin (0.8 U/mL). Clots were allowed to retract numerous times at 37 °C and photographed. Quantification was carried out by measuring clot areas using Image J 1.8.0 software (National Institute of Mental Health, Bethesda, MD, USA). Percentage clot retraction was calculated using:

Retraction (%) = 100 − [(sample clot area/intact sample area) × 100](2)

### 4.10. Immunoblotting Analysis

After platelet aggregation, the reaction was stopped by adding protease and phosphatase inhibitor-contained RIPA lysis buffer (50 mM pH 7.5 Tris-HCl, 150 mM NaCl, 1% Triton X-100, 1% sodium deoxycholate, 0.1% SDS and 2 mM EDTA). Protein concentrations were determined using a BCA assay. Equal amounts of proteins from platelet lysates were separated by SDS-polyacrylamide gel electrophoresis and transferred to PVDF membranes, which were then blocked with 5% skim milk in TBS buffer. Membranes were reacted with primary antibodies overnight at 4 °C and with secondary antibodies for 2 h at RT. Protein bands were visualized using a chemiluminescent substrate and photographed using a LAS-4000 (Fujifilm, Tokyo, Japan) luminescent image analyzer.

### 4.11. Statistical Analysis

Analysis of variance (ANOVA) was used to determine the significances of intergroup differences, and when ANOVA indicated a significant difference, groups were further compared using Tukey’s post hoc test in SPSS V20.0 software (SPSS, Inc., Chicago, IL, USA). Results are presented as means ± standard deviations and numbers of observations. *p* values of <0.05 were considered statistically significant.

## 5. Conclusions

Our study indicates that daidzein inhibits collagen-induced platelet aggregation by activating cAMP and inhibiting TXA_2_ production and the PI3K/Akt and MAPK p38/ERK signaling pathways. This inhibition of platelet activation by daidzein reduces granule release and fibrinogen binding to integrin α_IIb_β_3_ and, ultimately, inhibits platelet-mediated clot retraction. Taken together, these findings suggest that daidzein may have therapeutic potential as an anti-platelet and anti-thrombotic agent. However, further research is needed to determine the optimal dose and potential side effects of daidzein as a therapeutic agent and its efficacy and safety in clinical settings.

## Data Availability

The public available databases analyzed during the current study are included in the article, further inquiries can be directed to the corresponding author on reasonable request.
